# Prospective evaluation and classification of endoscopic findings for ureteral calculi

**DOI:** 10.1038/s41598-020-69158-w

**Published:** 2020-07-23

**Authors:** Shuzo Hamamoto, Shinsuke Okada, Takaaki Inoue, Teruaki Sugino, Rei Unno, Kazumi Taguchi, Ryosuke Ando, Atsushi Okada, Hiroyasu Miura, Tadashi Matsuda, Takahiro Yasui

**Affiliations:** 1 Department of Nephro-urology, Nagoya City University Graduate School of Medical Sciences, Nagoya, Japan; 2 Department of Urology, Gyotoku General Hospital, Hongyotoku 5525-2, Ichikawa City, Chiba Japan; 3 Department of Urology, Hara Genitourinary Hospital, Hyogo, Japan; 4 Department of Urology, Hachinohe Koyo Clinic, Aomori, Japan; 5 Department of Urology and Andrology, Kansai Medical University, Osaka, Japan

**Keywords:** Medical research, Risk factors, Signs and symptoms, Urology

## Abstract

Difficulty in performing ureteroscopic lithotripsy (URSL) depends on endoscopic findings surrounding calculi. In this multicentre prospective cohort study of 185 patients with a single ureteral stone who underwent ureteroscopic lithotripsy registered in the SMART study between January 2014 and February 2017, we established a classification of endoscopic findings and analysed risk factors for ureteral changes. We evaluated endoscopic findings (oedema, polyps, ureteral mucosa-stone adherence, and distal ureteric tightness) based on the SMART classification. Operative time and ureteral injuries were significantly correlated with endoscopic finding grades. Multivariate analyses revealed that mucosa-stone adherence (MSA) was strongly affected by hydronephrosis grade (odds ratio, 12.4; *p* = 0.022) and the interval before surgery (odds ratio, 1.10; *p* = 0.012). The cutoff value for MSA was 98 days, with a predictive accuracy of 0.78. Risk factors for distal ureteric tightness were age (odds ratio, 0.96; *p* = 0.004) and early intervention (odds ratio, 0.90; *p* = 0.023). The cutoff value was 34 days, with a predictive accuracy of 0.72. In conclusion, appropriate intervention around 34 days (limited to 98 days) after symptom onset is necessary for treating ureteral calculi. Even if intervention passed 98 days post-symptom onset, staged URSL, alternative procedures, and detailed informed consent should be planned in advance, assuming strong MSA.

## Introduction

The primary treatment for most ureteral calculi is fragmentation using shock wave lithotripsy (SWL) or ureteroscopic lithotripsy (URSL)^1^. Although both techniques have low complication rates, advances in endoscopic technology and superior stone-free (SF) rates for URSL have led to broader therapeutic indications^2–4^.

Impacted stones are often difficult to treat, even using flexible ureteroscopy (fURS)^5^, because they cause various pathological changes to the ureter, including oedematous mucosa, fibroepithelial polyps, and strong adhesion between the mucosa and the calculus^6^. Stone impaction results in chronic inflammation, eventually leading to these changes^7^.

However, no report has classified these endoscopic findings (EFs), and risk factors and their development remain unclear. In this study, we prospectively evaluated EFs during URSL, established a classification of EFs at the ureteral stone site, and analysed the risk factors for ureteral changes.

## Results

### Patient and stone characteristics

Patient characteristics are summarized in Table 1. The study included 142 men (76.7%) and 43 women (23.3%) with a mean age of 53.1 ± 14.1; 67 patients (36.2%) were treated within 30 days, and 73 patients (39.4%) waited for at least 61 days until treatment. Thirty-eight patients (20.5) had positive urine cultures and were treated with antibiotics.

**Table 1:** Patient and Stone characteristics (n = 185).

Age (years)	53.1 ± 14.1	
Sex; male (%)		142 (76.7)
BMI (kg/m^2^)	24.7 ± 5.4	
**ASA**		
I		112 (60.5)
II		67 (36.3)
III		6 (3.2)
**Comorbidity**		
Hypertension		57 (30.8)
Diabetes mellitus		29 (15.7)
Hyperlipidemia		25 (13.5)
**Symptom of colic pain**		
No		69 (37.3)
Yes		116 (62.7)
Stone side; right (%)		86 (46.5)
Stone diameter (mm)	8.3 ± 3.4	
Stone volume (mm^2^)	192.5 ± 202.4	
CT attenuation (HU)	893.9 ± 309.4	
**Stone location**		
Proximal		91 (49.2)
Middle		29 (15.7)
Distal		65 (35.1)
**Hydronephrosis**		
Grade 0		43 (23.2)
Grade 1		54 (29.2)
Grade 2		71 (38.4)
Grade 3		17 (9.2)
**Period until intervention (days)**		
0–30		67 (36.2)
31–60		45 (24.3)
≧61		73 (39.4)
**Preoperative urine culture**		
Negative		147 (79.5)
Positive		38 (20.5)

Data are given in means and standard deviations.

### Surgical outcomes

Among 185 cases, five patients had grade 3 distal ureteric tightness (DUT) due to which a semi-rigid ureteroscope (r-URS) could not be passed; therefore, we discontinued the procedure. Table 2 shows the surgical outcomes. The average operative time was 48.0 ± 33.7 min. SF status was maintained in 169 patients (93.4%) at one month postoperatively. Regarding to complications, postoperative fever was identified in three patients (1.7%). There were ureteral injuries in 43 patients, including 33 patients at the stone site and 10 patients due to ureteral access sheath (UAS) placement. Grade 1, 2, and 3 ureteral injuries at the stone site were identified in 27 (15.0%), 4 (2.2%), and 2 (1.1%) patients. Grade 1, 2, and 3 ureteral injuries due to UAS placement were identified in 7 (3.9%), 2 (1.1%), and 1 (0.6%) patients, respectively.

**Table 2:** Surgical outcomes (n = 180).

Ureteral access sheath, n (%)	Not use	67 (37.2)
	9.5/11.5Fr	33 (18.3)
	10/12Fr	8 (4.5)
	11/13Fr	36 (20.0)
	12/14Fr	36 (20.0)
Operative time (min)	48.0 ± 33.7	
**Stone free, n (%)**		169 (93.4)
Ureteral injury at the stone site, n (%)	G1 (mucosa)	27 (15.0)
	G2 (muscle)	4 (2.2)
	G3 (fat)	2 (1.1)
Ureteral injury for UAS placement, n (%)	G1 (mucosa)	7 (3.9)
	G2 (muscle)	2 (1.1)
	G3 (fat)	1 (0.6)
Postoperative fever		3 (1.7)

*UAS* ureteral access sheath.

### Incidence of endoscopic changes and association between endoscopic findings and time interval until intervention

In total, 83 (46.1%) and 34 (18.9%) patients had grade 1 and 2 oedematous changes at the stone site, respectively; 35 (19.4%) patients had grade 1 polyps. Regarding the ureteral MSA, 53 (29.4%) and 17 (9.4%) patients had grade 1 and 2 EFs, respectively. With respect to DUT, 52 (28.1%), 24 (13.0%), and 5 (2.7%) patients had grade 1, 2, and 3 DUT, respectively. Incidence of oedema and polyps were not associated with the time interval until intervention. However, the incidence of MSA increased significantly with prolonged time interval until intervention (*p* = 0.045). However, the incidence of DUT decreased significantly with prolonged time interval until intervention (*p* = 0.009) (Table 3).

**Table 3:** Association between endoscopic findings and period until intervention.

	Total n (%)	Period until intervention (days)			*p* value
		0–30	31–60	≧61	
**Oedema**					0.475
G0	63 (35.0)	22 (34.4)	12 (27.3)	29 (40.3)	
G1	83 (46.1)	30 (46.9)	25 (56.8)	28 (38.9)	
G2	34 (18.9)	12 (18.7)	7 (15.9)	15 (20.8)	
**Polyps**					0.700
G0	145 (80.6)	52 (81.2)	37 (84.1)	56 (77.8)	
G1	35 (19.4)	12 (18.8)	7 (15.9)	16 (22.2)	
**Mucosa-stone adherence (MSA)**					0.045
G0	110 (61.1)	46 (71.9)	27 (61.4)	37 (51.4)	
G1	53 (29.4)	16 (25.0)	14 (31.8)	23 (31.9)	
G2	17 (9.4)	2 (3.1)	3 (6.8)	12 (16.7)	
**Distal ureteral tightness (DUT)**					0.009
G0	104 (56.2)	34 (50.7)	25 (55.6)	45 (61.6)	
G1	52 (28.1)	15 (22.4)	12 (26.7)	25 (34.2)	
G2	24 (13.0)	15 (22.4)	7 (15.5)	2 (2.8)	
G3	5 (2.7)	3 (4.5)	1 (2.2)	1 (1.4)	

### Association between surgical outcomes and endoscopic findings

Operative time was prolonged significantly as the grade level increased for oedema (*p* < 0.001), polyps (*p* = 0.005), and MSA (*p* < 0.001). With respect to SFR, there were no significant differences in terms of grade levels between these EFs. In contrast, patients with grade 3 DUT had significantly lower SFR because of the impossibility of UAS insertion. With regard to ureteral injuries, the incidence and degree of ureteral damage at the stone site increased significantly with increased EF grade, including oedema (*p* < 0.001), polyp (*p* < 0.001), and MSA (*p* < 0.001). Significant association between ureteral injuries due to UAS placement and EF grade was not detected. These results are also shown in Table 4.

**Table 4:** Association between surgical outcomes and endoscopic findings.

	Oedema				Polyps			Mucosa-stone adherence (MSA)				Distal ureteric tightness (DUT)				
	G0	G1	G2	*p* value	G0	G1	*p* value	G0	G1	G2	*p* value	G0	G1	G2	G3	*p* value
n (%)	63 (35.0)	83 (46.1)	34 (18.9)		145 (80.6)	35 (19.4)		110 (61.1)	53 (29.4)	17 (9.4)		104 (56.2)	52 (28.1)	24 (13.0)	5 (2.7)	
Operative time (minutes)	39.8 ± 23.3	42.0 ± 21.9	77.8 ± 53.3	< 0.001	45.0 ± 33.1	60.5 ± 34.2	0.005	39.4 ± 21.7	56.6 ± 41.7	76.7 ± 47.7	< 0.001	51.5 ± 39.6	43.5 ± 22.8	42.3 ± 23.1	17.2 ± 4.0	0.253
Stone free, n (%)	59 (93.6)	77 (92.7)	33 (97.0)	0.846	137 (94.5)	32 (91.4)	0.449	104 (94.5)	51 (96.2)	14 (82.4)	0.110	96 (92.3)	50 (96.1)	23 (95.8)	0 (0)	< 0.001
Ureteral injury at the stone site, n (%)				< 0.001			< 0.001				< 0.001					0.666
G0 (no injury)	52 (82.5)	76 (91.6)	20 (58.8)		126 (86.9)	21 (22.2)		97 (88.2)	42 (79.2)	8 (47.1)		86 (82.7)	40 (77.0)	21 (87.5)	5 (100)	
G1 (mucosa)	10 (15.9)	7 (8.4)	10 (29.4)		16 (11.0)	11 (34.2)		12 (11.8)	10 (18.8)	5 (29.4)		13 (12.5)	11 (21.1)	3 (12.5)	0 (0)	
G2 (muscle)	1 (1.6)	1 (0)	2 (5.9)		2 (1.4)	2 (8.6)		1 (0.9)	1 (1.9)	2 (11.8)		4 (3.8)	0 (0)	0 (0)	0 (0)	
G3 (fat)	0 (0)	0 (0)	2 (5.9)		1 (0.7)	1 (5.7)		0 (0)	0 (0)	2 (11.8)		1 (1.0)	1 (1.9)	0 (0)	0 (0)	
Ureteral injury for UAS placement, n (%)				0.139			0.136				0.874					0.929
G0 (no injury)	58 (92.0)	79 (95.1)	32 (94.1)		138 (95.2)	32 (91.4)		104 (94.6)	50 (94.3)	16 (94.1)		97 (93.3)	50 (96.2)	23 (95.8)	5 (100)	
G1 (mucosa)	4 (22.2)	3 (3.6)	0 (0)		6 (4.1)	1 (2.9)		4 (3.6)	2 (3.8)	1 (5.9)		5 (4.8)	1 (1.9)	1 (4.2)	0 (0)	
G2 (muscle)	1 (3.2)	0 (0)	1 (2.9)		1 (0.7)	1 (2.9)		1 (0.9)	1 (1.9)	0 (0)		1 (1.0)	1 (1.9)	0 (0)	0 (0)	
G3 (fat)	0 (0)	0 (0)	1 (2.9)		0 (0)	1 (2.9)		1 (0.9)	0 (0)	0 (0)		1 (1.0)	0 (0)	0 (0)	0 (0)	

Data are given in means and standard deviations.

*UAS* ureteral access sheath.

### Predictive factors for prolonged surgical time

We analysed the predictive factors for prolonged operative times of more than 90 min (Table 5). Univariate analysis indicated that factors were significantly associated with prolonged operative time, included stone size, grade-2 oedema, and presence of polyps or MSA. Using multivariate logistic regression analysis, we found that oedema (grade 0 vs. 2: OR 28.3; *p* = 0.011), and MSA (grade 0 vs. 1: OR 6.34; *p* = 0.038, grade 0 vs. 2: OR 16.3; *p* = 0.009) were independent predictors for prolonged URSL.

**Table 5:** Predictive factors of prolonged operative time (≥ 90 min).

Variable	Univariate			Multivariate		
	OR	(95% CI)	*p* value	OR	(95% CI)	*p* value
Age (years)	1.02	(0.98–1.06)	0.264	1.06	(0.98–1.13)	0.057
Sex (male vs. female)	1.75	(0.61–4.99)	0.295	0.79	(0.15–4.13)	0.780
BMI (≤ 25 vs. > 25 kg/mm^2^)	0.52	(0.18–1.52)	0.232	1.11	(0.27–4.53)	0.881
Stone size (mm^2^)	1.02	(1.01–1.03)	< 0.001	1.01	(0.98–1.03)	0.462
CT attenuation (HU)	1.00	(0.99–1.00)	0.917	1.00	(0.99–1.00)	0.774
**Stone location**						
Proximal ureter	1.00	(Reference)		1.00	(Reference)	
Middle ureter	2.01	(0.66–6.12)	0.220	2.42	(0.46–12.5)	0.293
Distal ureter	0.25	(0.05–0.18)	0.079	0.37	(0.04–2.89)	0.345
**Oedema**						
Grade 0	1.00	(Reference)		1.00	(Reference)	
Grade 1	3.14	(0.34–28.8)	0.312	1.13	(0.09–13.0)	0.922
Grade 2	38.4	(4.73–311.0)	< 0.001	28.3	(2.13–374.0)	0.011
Polyps (grade 0 vs. 1)	3.05	(1.09–8.54)	0.034	0.31	(0.05–1.73)	0.181
**Mucosa-stone adherence**						
Grade 0	1.00	(Reference)		1.00	(Reference)	
Grade 1	6.34	(1.61–25.0)	< 0.001	6.34	(1.10–36.4)	0.038
Grade 2	25.0	(5.57–112.0)	< 0.001	16.3	(1.97–134.0)	0.009

### Preoperative factors Influencing EFs

Univariate analysis is shown in Table 6a. Patients with ureteral oedematous changes were more likely to have large stones. Patients who had polyps at the stone site were more likely to be females, have positive urine cultures, larger stones, and stones located in the middle ureter and distal ureter, as well as grade > 2 hydronephrosis. Patients who had grade-2 MSA were more likely to be women, have hyperlipidaemia, grade > 2 hydronephrosis, and longer time interval until surgery. Patients who had grade > 2 DUT were more likely to be younger and have shorter time interval until surgery.

**Table 6:** Parameters associated with severe endoscopic findings.

	Oedema (grade2)			Polyp (grade1)			MSA (grade2)			DUT (grade > 2)		
	OR	(95 %CI)	*p* value	OR	(95 %CI)	*p* value	OR	(95 %CI)	*p* value	OR	(95 %CI)	*p* value
**(a) Univariate analyses**												
Age (years)	0.98	(0.95–1.00)	0.107	0.99	(0.97–1.02)	0.808	1.01	(0.97–1.05)	0.564	0.96	(0.92–0.98)	0.004
Sex (male vs. female)	1.23	(0.52–2.90)	0.630	2.36	(1.06–5.25)	0.034	2.72	(1.01–7.28)	0.047	0.82	(0.23–1.81)	0.407
BMI (≤ 25 vs. > 25 kg/mm^2^)	0.77	(0.35–1.66)	0.506	0.84	(0.39–1.79)	0.658	0.74	(0.27–1.98)	0.540	1.10	(0.49–2.46)	0.801
**Comorbidity**												
Hypertension	0.65	(0.27–1.54)	0.326	1.24	(0.56–2.71)	0.594	1.37	(0.51–3.70)	0.531	0.53	(0.20–1.40)	0.204
Hyperlipidemia	1.84	(0.70–4.85)	0.215	2.23	(0.87–5.70)	0.093	3.45	(1.17–10.2)	0.024	0.20	(0.02–1.51)	0.118
Diabetes mellitus	1.83	(0.73–4.58)	0.196	1.40	(0.54–3.59)	0.487	2.77	(0.95–8.02)	0.060	0.35	(0.79–1.58)	0.173
Urine culture (negative vs. positive)	0.80	(0.30–2.09)	0.642	2.50	(1.10–5.69)	0.028	2.55	(0.92–7.02)	0.072	0.77	(0.27–2.19)	0.633
**Stone size (mm**^**2**^)												
≤ 25	1.00	(Reference)		1.00	(Reference)		1.00	(Reference)		1.00	(Reference)	
25–50	1.93	(0.55–6.78)	0.305	3.24	(1.10–9.57)	0.033	4.58	(0.94–22.1)	0.058	0.46	(0.17–1.20)	0.112
≥ 50	8.90	(2.82–28.0)	< 0.001	3.95	(1.33–11.8)	0.014	4.75	(0.96–23.4)	0.055	0.46	(0.17–1.24)	0.125
**Stone location**												
Proximal ureter	1.00	(Reference)		1.00	(Reference)		1.00	(Reference)		1.00	(Reference)	
Middle ureter	1.83	(0.68–4.90)	0.230	3.34	(1.38–8.09)	0.007	1.80	(0.63–5.09)	0.268	0.26	(0.05–1.20)	0.084
Distal ureter	1.00	(0.42–2.34)	0.993	0.28	(0.08–0.863)	0.027	–			0.43	(0.16–1.08)	0.073
Hydronephrosis (grade 0/1 vs. 2/3)	2.01	(0.93–4.33)	0.073	2.84	(1.29–6.23)	0.009	11.3	(2.53–50.7)	0.001	1.19	(0.53–2.62)	0.671
Period until intervention (week)	1.01	(0.98–1.04)	0.288	1.02	(0.99–1.04)	0.217	1.05	(1.02–1.09)	0.002	0.90	(0.83–0.97)	0.009
**(b) Multivariate analyses**												
Age (years)	0.97	(0.94–1.00)	0.089	0.99	(0.96–1.03)	0.921	1.02	(0.97–1.07)	0.498	0.96	(0.92–0.99)	0.020
Sex (male vs. female)	1.56	(0.53–4.60)	0.421	2.36	(1.06–5.25)	0.303	1.19	(0.26–5.33)	0.820	0.80	(0.23–2.65)	0.710
BMI (≤ 25 vs. > 25 kg/mm^2^)	0.49	(0.19–1.27)	0.145	0.71	(0.28–1.76)	0.464	0.77	(0.19–3.10)	0.719	1.14	(0.46–2.82)	0.775
**Comorbidity**												
Hypertension	0.49	(0.14–1.71)	0.264	0.78	(0.27–2.24)	0.644	0.35	(0.06–2.02)	0.244	1.21	(0.37–3.92)	0.751
Hyperlipidemia	2.35	(0.56–9.66)	0.238	1.89	(0.52–6.88)	0.334	3.52	(0.46–26.6)	0.223	0.28	(0.02–2.80)	0.281
Diabetes mellitus	1.42	(0.41–4.91)	0.580	0.76	(0.22–2.58)	0.668	1.83	(0.31–10.5)	0.499	1.25	(0.22–7.09)	0.798
Urine culture (negative vs. positive)	0.94	(0.29–3.00)	0.912	1.76	(0.64–4.80)	0.272	1.35	(0.30–6.05)	0.697	1.00	(0.28–3.57)	0.994
**Stone size (mm**^**2**^)												
≤ 25	1.00	(Reference)		1.00	(Reference)		1.00	(Reference)		1.00	(Reference)	
25–50	1.52	(0.38–5.94)	0.547	1.94	(0.59–6.38)	0.272	1.70	(0.25–11.1)	0.581	0.62	(0.20–1.85)	0.388
≥ 50	9.50	(2.68–33.7)	< 0.001	2.27	(0.68–7.53)	0.179	0.78	(0.11–5.53)	0.807	0.55	(0.17–1.72)	0.305
**Stone location**												
Proximal ureter	1.00	(Reference)		1.00	(Reference)		1.00	(Reference)		1.00	(Reference)	
Middle ureter	1.50	(0.48–4.64)	0.483	3.24	(1.25–8.40)	0.015	1.92	(0.50–7.25)	0.338	0.29	(0.05–1.46)	0.133
Distal ureter	2.40	(0.79–7.22)	0.119	0.37	(0.11–1.30)	0.123	–			0.47	(0.16–1.35)	0.161
Hydronephrosis (grade 0/1 vs. 2/3)	2.04	(0.77–5.40)	0.152	1.58	(0.64–3.89)	0.319	12.4	(1.43–107.0)	0.022	0.95	(0.37–2.43)	0.913
Period until intervention (week)	1.01	(0.97–1.04)	0.622	1.02	(0.98–1.05)	0.292	1.10	(1.02–1.19)	0.012	0.90	(0.83–0.98)	0.023

**Figure 1:**
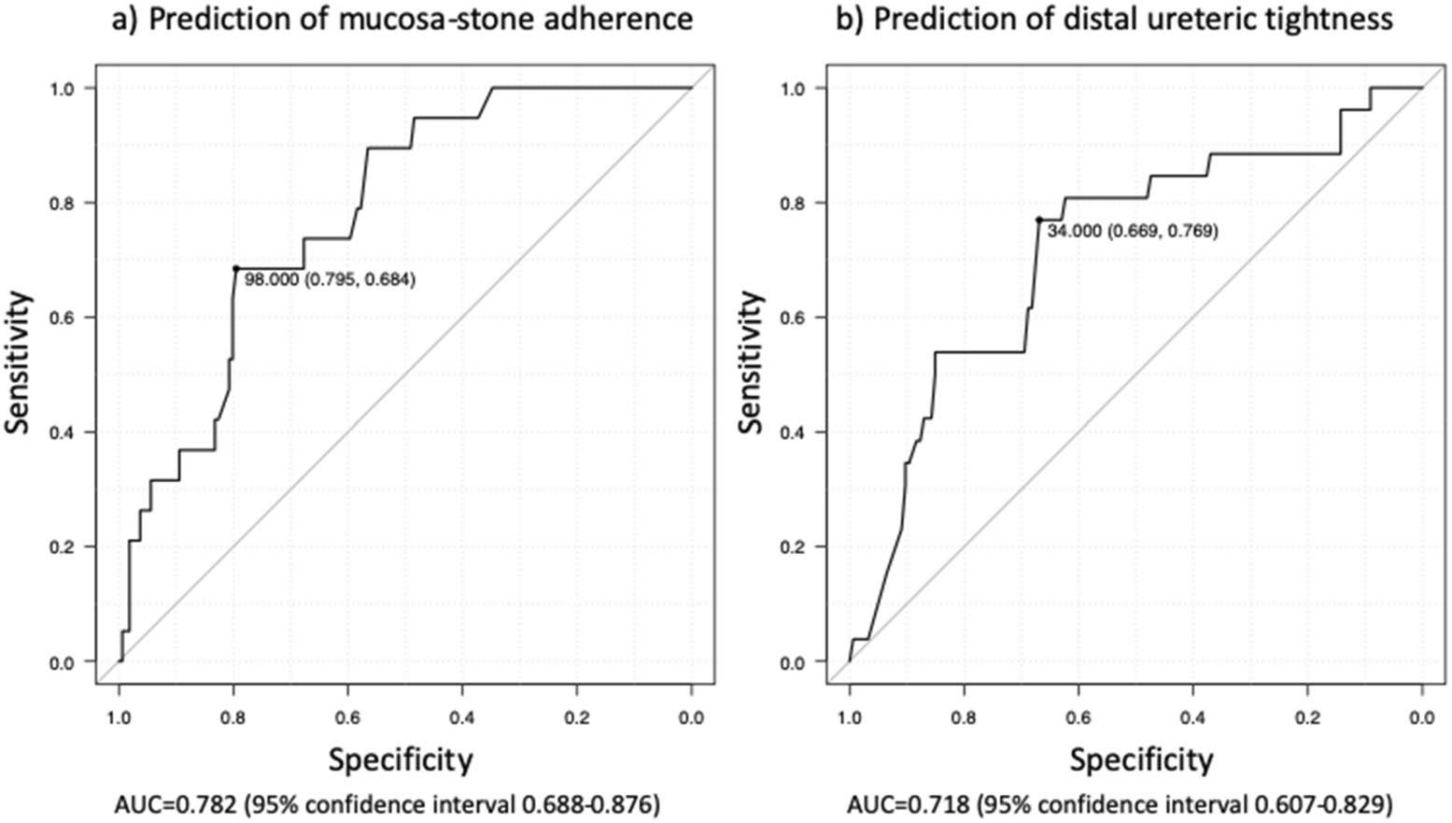
Receiver operating characteristic (ROC) curve of the duration until surgery for predicting mucosa-stone adherence (**a**) and distal ureteric tightness (**b**).

Multivariate analysis is shown in Table 6b. Large stone size (≤ 25 mm^2^ vs. ≥ 50 mm^2^; OR 9.50; *p* < 0.001) was an independent risk factor for the formation of oedema. Stone location (proximal vs. middle ureter: OR 3.24; *p* = 0.015) was an independent risk factor predicting polyp formation. Severe hydronephrosis (grade 0 or 1 vs. 2 or 3: OR 12.4; *p* = 0.022) and prolonged time interval until surgery (OR, 1.10; *p* = 0.012) were independent factors for the development of MSA to ureteral mucosa. Receiver operating characteristic (ROC) curve analysis revealed that 98 days until surgery was the optimal cutoff value predictive for MSA, with an area under the curve of 0.78 (95% confidence interval, 0.69–0.88) (Fig. 1a). This cutoff had a sensitivity of 68.4% and a specificity of 79.5%. Younger age (OR 0.96; *p* = 0.004) and immediate intervention (OR 0.90; *p* = 0.023) were independent risk factors predicting grade > 2 DUT. ROC curve analysis revealed that 34 days was the cutoff value predictive for DUT, with an area under the curve of 0.72 (95% confidence interval, 0.61–0.83) (Fig. 1b). This cutoff had a sensitivity of 76.9% and a specificity of 66.9%).

## Discussion

In this multicentre prospective cohort study, we proposed the Skilled Endoscopic Management of Renal and Ureteral Stones (SMART) classification of ureteral changes based on EFs. To the best of our knowledge, this is the first study to classify EFs in this scenario and use the SMART classification to predict surgical difficulty. This classification worked well for evaluating EFs in terms of the association between grades of EFs , operative times, and intraoperative ureteral injuries at the stone site. Kuroda et al. reported that stone volume, HUs, operator experience, gender, preoperative stenting, and UAS diameter predicted operative times for fURSL^8^. However, that study did not include intraoperative EFs. We found that EFs but not stone volume, including oedema and MSA, were independent predictors for prolonged URSL (more than 90 min). If oedema and polyps prevent a clear view of the calculi, retrograde pressure irrigation and careful laser manipulation are needed to avoid miss-shooting. Embedded stones in the ureteral mucosa require time to be peeled off using URS. It is important to accurately predict operative time, because a longer surgical time represents a risk factor for severe complications^9,10^.

Patients with grade 3 DUT had significantly lower SF rates due to failure of URS insertion. Ito et al. found that stone volume and cumulative stone diameter were independent predictors for SF status after single fURSL^11^. However, this did not include intraoperative EFs. If a r-URS or UAS cannot be inserted into the stone site through the distal ureter, it is difficult to achieve SF status without complications^12^.

URSL for impacted stones showed lower SFR and higher intraoperative complications than that for non-impacted stones^5,13,14^. It is important to accurately predict impacted stones and ureteral condition at their surroundings to achieve SF status without complications. There are some reports analysing predictors for stone impaction. However, the results were not consistent because of varying definitions of impacted stones in practice^5,7,14,15^. Legemate et al. reported that female sex, ASA-score > 1, positive urine culture, prior stone treatment, and large stone burdens were predictors for impacted ureteral stones^5^. We previously reported that younger age, stones located at the middle ureter, and ureteral wall thickness (UWT) as measured at the stone site using preoperative non-contrast computed tomography^14^ were independent predictors, and a UWT of 3.49 mm was the optimal predictor for cutoff value. We also reported that higher UWT was associated with unfavourable EFs, including oedema, polyps, and MSA^14^.

We found that large stone size was a factor influencing the formation of oedema. Ureteral oedema may arise from ischemia secondary to chronic pressure or may be an immunological reaction to the stone material^7,16^. Large stones might be caused by chronic ischemia because pressure on the ureteral wall leads to the formation of oedema. Because some oedemas might continue to grow, large stones should be treated as soon as possible.

Fibroepithelial polyps may form as a result of repeated inflammation of ureteral tissues^17^. Idiopathic ureteral polyps develop most frequently in the proximal ureter^18^. Nevertheless, we found that stones located at the middle ureter were risk factors for the formation of polyps. This result was also shown in our previous report^14^. With respect to polyps associated with urolithiasis, anatomic narrowing, with the ureteral crossing the iliac artery, may lead not only to disturbing spontaneous stone passage, but also chronic inflammation.

With respect to MSA, it is important to remove embedded stones completely, because residual stone fragments may provoke inflammation, leading to stricture formation^19^. In the present study, the independent risk factors were severe hydronephrosis and prolonged time interval until surgery, with a cutoff value of 98 days. The mechanism of the formation of MSA has not been elucidated clearly. However, a chronic ureteral inflammatory reaction caused by the immobility of stones and persistent irritation may induce the submucosal migration and epithelisation over the stones. We speculated that once the impacted stone in the ureter led to hydronephrosis and a tortuous ureter, the chronic inflammatory reaction around the stone might worsen with increased ureteral peristalsis. Finally, it would induce MSA, which might lead to more severe hydronephrosis with a longer time interval until surgery. Thus, in patients suspected of severe adherence with a more than three-month time interval after symptom onset or with severe hydronephrosis, retrograde URSL with nephrostomy or percutaneous antegrade URSL may be better alternatives^20,21^.

It is important to observe distal ureteral findings at the beginning of URSL to choose the size of UAS. Traxer et al. reported that 13.3% of patients had severe ureteral injuries due to 12/14Fr UAS placement^22^. In contrast, in this study, 1.7% of patients had severe ureteral injuries due to UAS placement because we chose the UAS size according to DUT grade. In another report, 16% of urologists expressed concerns regarding the failure in UAS placement^23^. If a UAS is inserted into a narrow-caliber ureter with excessive bulking force, it may induce injury, avulsion, or secondary stricture. If a DUT is classified as grade 2 or 3, URSL should be performed without a UAS, or it can be terminated with stent placement and a staged procedure can be performed later. Recently, preoperative alpha blockade has been reported to reduce the maximal UAS insertion force and the consequent risk of ureteral injury. In addition, pre-stenting was a predictor for successful UAS insertion^24^. This is why pre-stenting and preoperative alpha blockade may improve distal ureteral findings. The causes of DUT development have not yet been elucidated. However, we found that independent factors for predicting DUT were younger age and shorter interval time until URSL, with a cutoff of 34 days. This might be associated with ureteral spasm due to renal colic. Koo et al. reported that men and younger patients required higher UAS insertion force because of ureteral narrowing^25^. Presumably, progressive expansion in ureteral diameter with aging may be associated with these results^26^, in addition to the loss of surrounding muscle mass in older patients. With regard to renal function, canine studies suggest that renal impairment may occur after 2 weeks of high-grade obstruction; thus, ureteral calculi should be treated as soon as possible. However, immediate intervention within one month may result in the impossibility of r-URS or UAS insertion.

This study had some limitations. We studied a relatively small cohort, limited to patients with a single ureteral stone without pre-stenting or pre-nephrostomy. Second, SMART classification was decided based on the operator’s subjective judgement. Almost all cases were evaluated by three investigators to make an accurate judgement. Finally, this was a preliminary study; therefore, external validation with a multicentre large prospective cohort will be needed.

In conclusion, we first defined EFs during URSL and used the SMART classification to predict surgical difficulty worldwide. Retention time of a ureteral stone and the presence of hydronephrosis were risk factors for MSA. There is a relationship between early intervention and patient age with DUT. URSL should be performed as soon as possible after 34 days following the symptom onset. However, for suspected stone impaction cases with more than 98 days after symptom onset, alternative procedures and detailed informed consent should be planned in advance.

## Methods

### Study population

**Figure 2:**
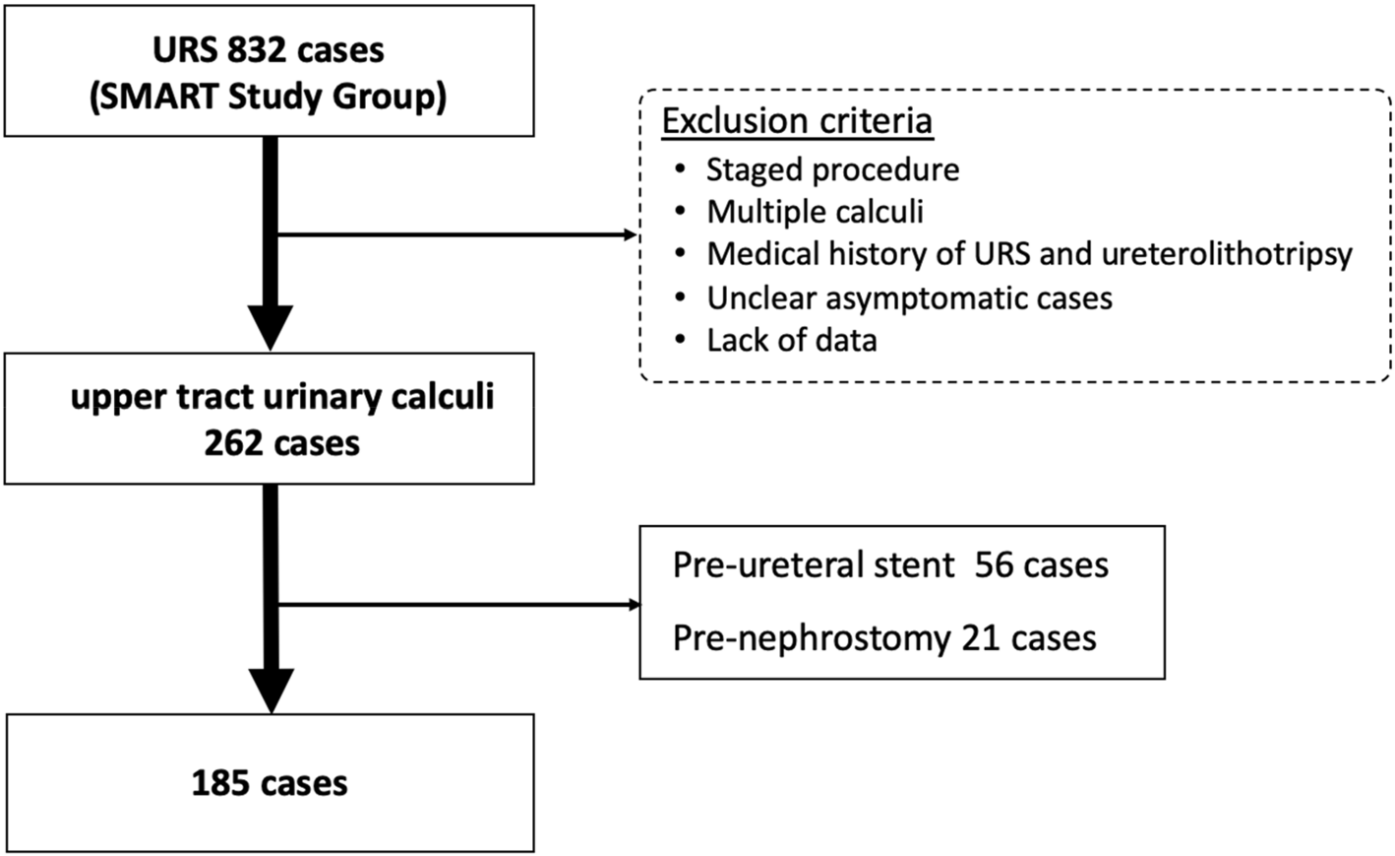
Flow chart of the study: Among 832 patients, 262 patients, who underwent a ureteroscopic lithotripsy (URSL) for a single ureteral stone, were analysed. All patients with planned staged procedures, multiple calculi, medical history of URS or URSL, or unclear duration until intervention were excluded. Patients who had received preoperative ureteral stent insertion (56 patents) or preoperative nephrostomy insertion (21 patients) were excluded as well. Finally, we analysed 185 patients in this study.

This prospective study was performed at three academic centres in the SMART study group. The study was conducted in accordance with the Helsinki Declaration. This study was also approved by the institutional review boards at Kansai Medical University (T24-5) and Toyota Kosei Hospital (24ST-04) before the start of the study, and selected patients who consented to participation. The study flow chart is shown in Fig. 2. Among 832 patients who underwent URSL for kidney and ureteral stones registered in the SMART Study Group between January 2014 and February 2017, we analysed 185 patients who underwent URSL for single ureteral stones. We excluded all patients undergoing planned staged procedures; those with multiple calculi, a medical history of URS or URSL, or an unclear date of when the stone passage stopped; and those who underwent preoperative ureteral stent insertion (56 patients) or preoperative nephrostomy insertion (21 patients). All patients provided written informed consent prior to inclusion. The surgeons recommended patients to be treated as soon as possible. However, the treatment timing was decided according to the patient’s desire, hospital capacity, and efficacy of medical expulsive therapy. As there were no reports analysing the association between the impacted stones and the time interval until surgery, we calculated the sample size based on our preliminary data. Our preliminary data showed that the ratio of grade 2 MSA of patients who were treated within 30 days and could not be treated within 60 days were 6.0% and 23.7% respectively. Calculated based on these data, 61 patients were required in each group with a significance level of 0.05% and a power of 80%. Totally, 183 patients were required, and in the present study we analysed the data from 185 patients.

### Data collection

Table 1 shows the collected data. Hydronephrosis (grades 0/1/2/3) was defined according to the Ellenbogen classification^27^. The duration until intervention was calculated as days from the identification of clinical signs related to stones to the day of surgery.

Preoperative evaluations included clinical examination and standard imaging (abdominal plain X-ray [KUB], ultrasound, and computed tomography) to determine the location and size of stones. Stone volume was estimated using the formula described by Tiselius and Andersson (length × width × 3.14 × 0.25)^28^. We usually perform pre-procedural urine cultures, and we would change the antibiotics based on the results of urine cultures. Postoperative fever was defined as > 38.5 degrees Celsius. Ureteral injury was graded according to Traxer’s classification^22^. SF was defined as not only endoscopic clearance at the end of the operation, but also as absence of stones or fragments > 2 mm on KUB and ultrasound one month after surgery.

### Surgical procedure

All URSL procedures were performed using a 6.4/7.8Fr (Olympus, Tokyo, Japan), 6.5/8.5Fr (Richard Wolf, Knittlingen, Germany), or 7.0/8.0Fr (Karl Storz, Tuttlingen, Germany) r-URS and fURS, including 7.5-F Flex-X2 (Karl Storz), 7.95-F URF-P6 (Olympus), or 8.5-F UFV-V2 (Olympus) under spinal or general anaesthesia.

At the beginning of the URSL, distal ureteral findings were observed using an r-URS. If the r-URS could not be inserted, the surgery was discontinued after inserting a ureteral stent. A UAS was placed in almost all patients except for those with distal ureteral stones. A ureteral access sheath of 9.5/11.5Fr, 10/12Fr, 11/13F, or 12/14 F sizes was placed in 113 patients (Table 3).

A holmium laser was used to fragment the target stone while securing a clear view of the endoscopic field using retrograde irrigation. ﻿Controlled irrigation with the single action pumping system (Boston Scientific, Natick, MA) was used for each case. Stone fragments were extracted until they completely disappeared using basket forceps and the one-surgeon basketing technique, as previously reported^29^. Selection of the type and size of device was based on the surgeon’s discretion under the surveillance of members of the SMART study group (SH, SO or TI). At the end of each procedure, the UAS was removed, and ureteral injuries were visually assessed. A ureteral stent was inserted at the end of each surgery and left in situ, generally for 1–4 weeks.

### EFs (SMART classifications)

**Figure 3:**
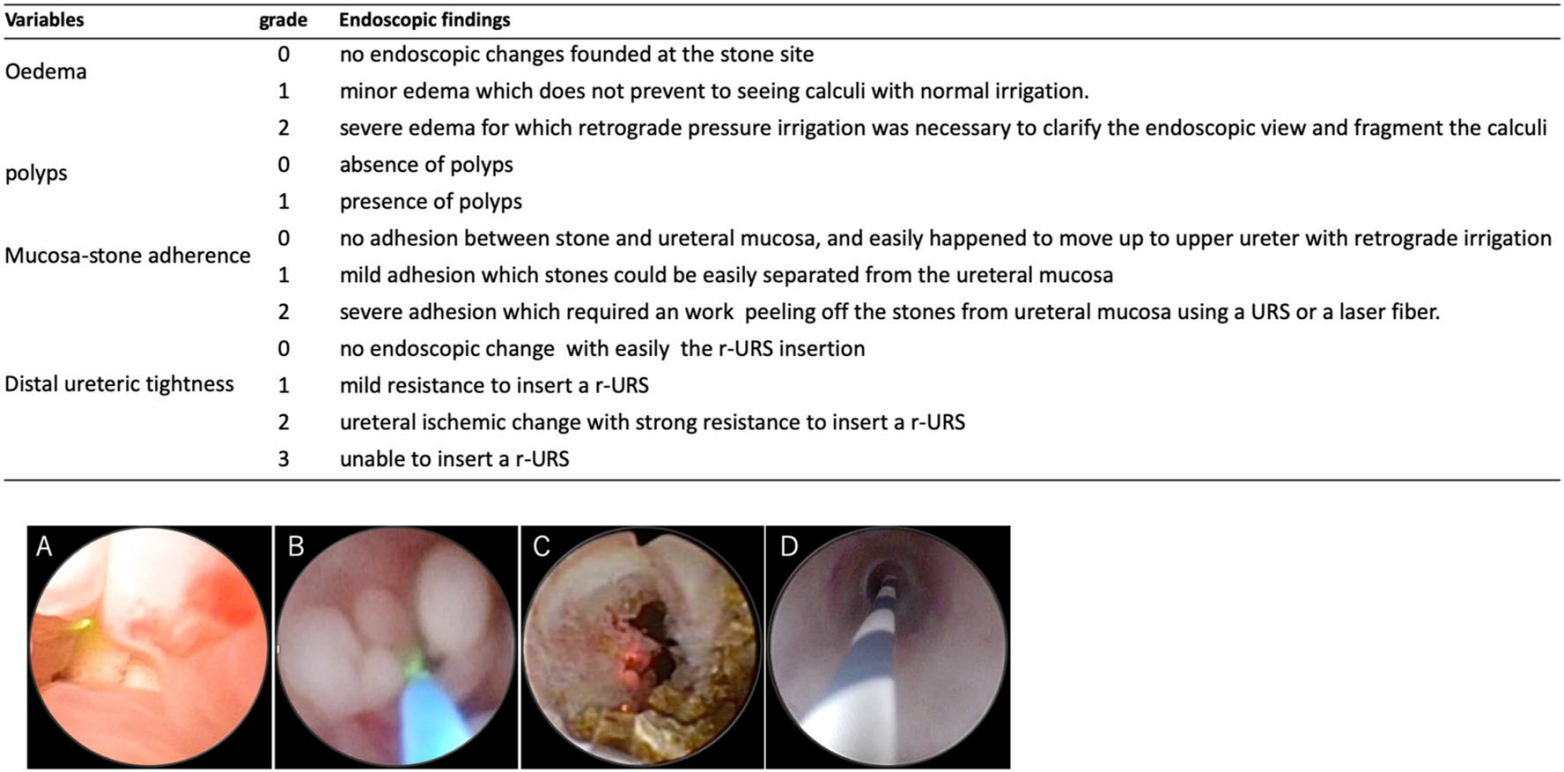
SMART classification of endoscopic findings and endoscopic views of ureteral changes (grade 2 oedema, polyps, mucosa-stone adherence (MSA), distal ureteric tightness (DUT)). Endoscopic views of ureteral changes are shown in (**A**)–(**D**). (**A**) grade 2 oedema, (**B**) grade 1 polyps, (**C**) grade 2 MSA, (**D**) grade 2 DUT.

We evaluated the EFs at the stone site (oedema, polyps, MSA) and DUT prospectively based on the SMART classification that was independently created for this study (Fig. 3). Grade 1 was defined as minor oedema that does not prevent seeing calculi with natural irrigation of about 100 cm saline. If retrograde pressure irrigation using a single action pumping system was necessary to clarify the endoscopic view and to fragment the calculi due to severe oedema, the oedema was graded as 2. Polyp grades were determined based on the presence or absence of polyps (grade 0 or 1). Grade 0 MSA classified stones that never stuck to the ureteral mucosa and easily moved up to the upper ureter with retrograde irrigation. G1 was defined as cases where stones easily separated from the ureteral mucosa; G2 cases were defined as those requiring removal using a URS or a laser fibre. Regarding DUT, if mild resistance to r-URS insertion was noted during observation of the lower ureter, the narrowing was graded as 1. G2 cases were defined as those involving strong resistance. In cases of difficulty in inserting r-URS, double guidewires were used to overcome these situations; however, a ureteral balloon catheter was not used in the present study. If r-URS could not be inserted due to severe DUT, the DUT was graded as 3. EFs were evaluated by three investigators (SH, SO or TI).

### Endpoints

The primary endpoint was incidence of EFs according to the periods until interventions. The secondary endpoint was to analyse an association between EFs and surgical outcomes including operative time, stone free rate, and ureteral injury. The additional endpoint was to analyse the predictive factors for prolonged surgical time and preoperative factors influencing EFs.

### Statistical analysis

Normally distributed variables are expressed as means ± standard deviations. Categorical variables are expressed as numbers (percentages). Clinical variables were compared using the Mann–Whitney U-test, one-way analysis of variance, and the Chi-square test. Univariate and multivariate analyses were performed using a logistic regression model. The duration cutoff values predicting stone adhesion or DUT and their predictive accuracies were determined using ROC curve analysis. Differences were considered statistically significant when *p* < 0.05. All statistical analyses were performed using EZR for R^30^ by two authors (SH and RA), then validated by the others.
